# Recent Advances in Allogeneic CAR-T Cells

**DOI:** 10.3390/biom10020263

**Published:** 2020-02-10

**Authors:** Dong Wook Kim, Je-Yoel Cho

**Affiliations:** Department of Biochemistry, BK21 PLUS Program for Creative Veterinary Science Research and Research Institute for Veterinary Science, College of Veterinary Medicine, Seoul National University, Seoul 151-742, Korea; bellocan@snu.ac.kr

**Keywords:** CAR-T, switch molecule, universal CAR-T, allogeneic, cancer

## Abstract

In recent decades, great advances have been made in the field of tumor treatment. Especially, cell-based therapy targeting tumor associated antigen (TAA) has developed tremendously. T cells were engineered to have the ability to attack tumor cells by generating CAR constructs consisting of genes encoding scFv, a co-stimulatory domain (CD28 or TNFRSF9), and CD247 signaling domains for T cell proliferation and activation. Principally, CAR-T cells are activated by recognizing TAA by scFv on the T cell surface, and then signaling domains inside cells connected by scFv are subsequently activated to induce downstream signaling pathways involving T cell proliferation, activation, and production of cytokines. Many efforts have been made to increase the efficacy and persistence and also to decrease T cell exhaustion. Overall, allogeneic and universal CAR-T generation has attracted much attention because of their wide and prompt usage for patients. In this review, we summarized the current techniques for generation of allogeneic and universal CAR-T cells along with their disadvantages and limitations that still need to be overcome.

## 1. Introduction

Known as a living drug, CAR-T cell therapy has been intensively performed over the past decade. Promising research data have been accumulated and more efficient and controllable CAR-T cells have been developed. At first, CAR-T therapy was tested on patients with hematological cancers. Especially, patients with B-cell Acute Lymphoblastic Leukemia (B-ALL) without improvement with known therapies such as hematopoietic stem cell transplantation (HSCT) and chemotherapies were tested on, since CAR-T therapy was the last treatment option. Currently, two CAR-T cell drugs, YESCARTA (axicabtagene ciloleucel) and KYMRIAH (tisagenlecleucel), are FDA approved to, respectively, treat adult patients with certain types of large B-cell lymphoma who have not responded to or who have relapsed after at least two other kinds of treatment and to treat patients up to 25 years of age with B-cell precursor ALL that is refractory or in second or later relapse. Three other CAR-T with an International Nonproprietary Name (INN), vadacabtagene leraleucel, idecabtagene vicleucel, and lisocabtagene maraleucel, are referenced in IMGT/mAb-DB, from IMGT^®^, the international ImMunoGeneTics information system^®^ [1]. Even though there are still side-effects during CAR-T therapy such as cytokine release syndrome (CRS) and neurotoxicity, recent studies using modified CAR-T cells through various advanced techniques showed promising results for using CAR-T cells more efficiently and safely. The efficient and safe usage of CAR-T cells may include the following concepts: (1) production of CAR-T cells before injecting them back into the patients should be done fast to avoid further progression of disease. (2) CAR-T cells could be used both allogeneically and universally. Gene editing is the most widely used technique to create universal CAR-T cells. A major target for this system is the T cell receptor (TR) [2] to minimize Graft-versus-Host Disease (GvHD) that normally occurs during allogeneic transplantation. To minimize GvHD, chemotherapy regimens including immunosuppressive combinations of Fludarabine and Cyclophosphamide, and serotherapy using Alemtuzumab (anti-CD52 mAb) could be administered ahead of allogeneic CAR-T treatment [3]. Another advantage of TR depleted CAR-T cells is that they can be used off-the-shelf. Even though autologous CAR-T therapy works well for GvHD, the time needed to prepare CAR-T cells using T cells obtained from the patients is too long and the disease may progress further before the CAR-T therapy can commence. Indeed, previous reports studying patients with diffuse large B-cell lymphoma or follicular lymphoma showed that 26% (10 of 38) of patients did not receive Tisagenlecleucel treatment due to the rapid disease progression [4]. In another study of children and young adults with B-cell lymphoblastic Leukemia, 7.6% (7 of 92) of patients also did not receive Tisagenlecleucel treatment due to death [5]. By preparing CAR-T cells for each disease in advance, it is possible to promptly use prepared CAR-T cells whenever needed. A further point to critically consider when using CAR-T cells as a drug is the careful handling of the side-effects. Since the curative effect of CAR-T cells stem mainly from various released cytokines, common side-effects are often associated with uncontrolled cytokine release and could be very harmful; for instance, some cytokines can penetrate the blood brain barrier (BBB) and cause neurotoxicity [6]. To prevent this problem, various safety switches have been developed such as the incorporation of suicide genes, expression of known target genes for therapeutic antibodies, and the addition of molecular switch proteins between CAR and tumor cells. In this review, we summarized the techniques used to generate allogeneic and universal CAR-T cells and discuss their advantages and considerations for their wide use.

## 2. General CAR Structure

**Figure 1:**
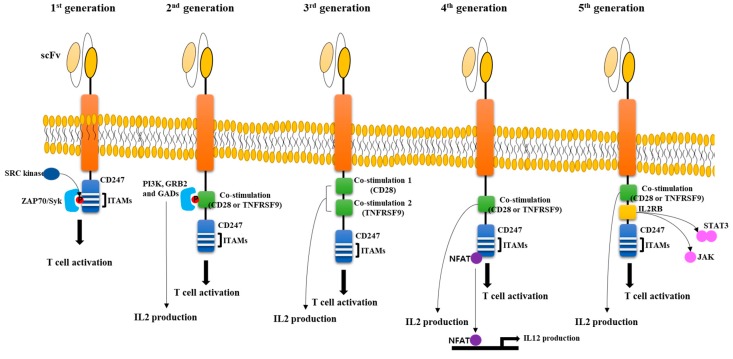
Structure of CAR-T cells. CAR-T cells is composed of single-chain variable fragment (scFv), transmembrane domain, and signaling domain. First CAR-T cells have only CD247 ITAM (immunoreceptor tyrosine-based activation motifs) domain, which cause activation of ZAP70/Syk tyrosine kinase, resulting in the activation of downstream signaling cascade. Second CAR-T cells have one additional co-stimulation domain derived from either of CD28 or TNFRSF9. Various proteins containing SH2 domain such as PI3K, GRB2, and GADs are recruited to co-stimulation domain and IL2 production is induced. In 3rd CAR-T cells, two co-stimulation domains are included, resulting in more cytotoxicity activities for tumor cells. In 4th CAR-T cells, additional gene for IL12 production is transduced into CAR-T cells. IL12 expression is controlled by NFAT transcription factor that is activated by binding to CD247 domain. This system is developed to activate innate immune system in case CAR-T cells does not recognize tumor cells due to low expression of antigen on the surface. In 5th CAR-T cells, JAK-STAT activation domain derived from IL2RB is incorporated between CD28 and CD247. This domain stimulates cell proliferation, prevents terminal differentiation, and shows better persistence.

The early trial to create CAR-T cells fused V_H_sp6 to Cα or Cβ, and found that V_H_Cα or V_H_Cβ chimeric chains can form heterodimers with β or α chains of the recipient T cell [7]. This was the first system to escape the MHC-restricted manner of T cell activation. The signaling domain of CAR has subsequently been engineered by adding a signaling domain of CD247 (First generation CAR), adding one co-stimulation domain either of TNFRSF9 or CD28 plus the CD247 domain (Second generation CAR), adding two co-stimulation domains of both CD28 and TNFRSF9 plus the CD247 domain (Third generation CAR), adding one co-stimulation plus the CD247 domain and IL12 expression system (Fourth generation CAR), and adding one co-stimulation, intracellular domain of a cytokine receptor (e.g., IL2RB chain fragment) binding STAT3 plus the CD247 domain (Fifth generation CAR) [8,9]. All modifications of CAR structures were made to enhance CAR-T cell activity, increase proliferation and cytokine release, and decrease T cell exhaustion. In theory, various modifications to the CAR structure are possible, but the basic structure used thus far may not be altered significantly. The general function and description of various signaling domains used in CAR structures are below and summarized in Figure 1.

### 2.1. CD247 Domain

CD247 (known as CD3ζ) is a transmembrane adaptor in the CD3 complex of TR co-receptor [10]. CD247 has three immunoreceptor tyrosine-based activation motifs (ITAMs), which are phosphorylated by SRC tyrosine kinase family and which recruit ZAP70/Syk tyrosine kinase, resulting in the activation of downstream signaling cascade for T cell activation [11,12,13,14]. Beside T cell activation, CD247 is also involved in the signaling of Nkp46/Nkp30 and is a low-affinity Fc receptor for IgG CD16 in NK cells [15,16]. For CAR-T construction, ITAMs are usually incorporated into the CAR structure to induce T cell proliferation and activation. The role of ITAM in CAR-T cell activation was identified to be mediated by heterodimerization with native CD247 partners inside cells. Furthermore, among the three ITAMs in CD247, the third one has been identified to not affect T cell activation, but the second one was identified as a critical factor for T cell activation [14].

### 2.2. TNFRSF9 Domain

Although successful trials with first generation CAR-T cells showed anti-tumor activity, the efficacy was insufficient due to the low T cell persistence. To overcome this, a co-stimulatory domain was added. TNFRSF9 (Known as 4-1BB and CD137) is a member of the tumor necrosis factor (TNF) receptor family and is known to be expressed in activated T cells [17]. The importance of a co-stimulatory domain for anti-tumor activity was demonstrated by comparing CAR-T cells with and without TNFRSF9 signaling domains targeting CD19. CAR-T cells with a TNFRSF9 domain showed powerful and specific cytotoxicity for B-ALL with more production of IL12 compared to CAR-T cells without a TNFRSF9 domain, indicating that an additional signaling domain is necessary for enhanced activity of CAR-T cells [18].

### 2.3. CD28 Domain

Another co-stimulatory domain for CAR-T activity is from CD28 and is known as a receptor for CD80 and to stimulate production of various interleukins upon T cell stimulation [19]. Phosphorylation at tyrosine 170 in the intracellular domain of CD28 is responsible for its binding to SH2-domain containing proteins such as PI3K, Grb2, and Gads, resulting in increased production of IL2 and activation of T cells [20,21,22]. Comparative analysis with various CAR constructs to see the effect of TNFRSF9 or CD28 co-stimulation domains identified a higher expansion of CAR-T cells with either co-stimulation domain than with the CD247 domain alone when cocultured with cancer cells expressing the target antigen. Furthermore, there is no significant difference between tumor lysis activity of CAR-T cells containing both a CD28 and TNFRSF9 domain and cells containing either of these domains alone [23], resulting in the 4th generation using either CD28 or TNFRSF9 alone.

### 2.4. IL12 Expression System

Adding a co-stimulatory domain seems to be sufficient for full CAR-T activity, but some studies showed that another factor may be needed to increase the tumor killing effect of CAR-T cells. A critical issue with using CAR-T cells is that they can only be activated when the antigen in the target tumor cell is exposed and captured, meaning some antigen-less tumor cells could escape from CAR-T cells. To overcome this, IL12 expressing CAR-T cells were developed and named as T cells redirected for universal cytokine-mediated killing (TRUCK) [24]. IL12 is known to be released from dendritic cells, macrophages, neutrophils, and B-lymphoblastoid cells through various stimulations [25,26,27]. Its main function includes differentiation of naïve T cells into Th1 cells, activation of natural killer cells and T lymphocytes, and anti-angiogenic activity [28,29,30]. IL12 released from activated CAR-T is deposited around the target tumor lesion before attracting innate immune cells such as natural killer cell and macrophages that can kill the tumor cells that are invisible to CAR-T cells [31,32]. The fourth generation CAR-T system utilizing an NFAT transcription factor to stimulate IL12 production has been tested on various tumors including solid tumors and showed promising results [33,34,35,36,37].

### 2.5. IL2RB Chain Fragment

Recently, newly developed fifth generation CAR-T cells, in which IL2RB chain fragments were inserted between the TR signaling (CD247) and co-stimulatory domains (CD28), was used to induce cytokine signaling. Since the IL2RB fragment has a STAT3 binding YXXQ motif, these CAR-T cells induced antigen-dependent activation of the JAK-STAT pathway, which promoted cell proliferation and prevented terminal differentiation. Also, this CAR-T cells showed better persistence and therapeutic effect on leukemia than CAR-T cells with a TNFRSF9 co-stimulation domain alone [38].

## 3. Effectiveness of Allogeneic CAR-T Cell Therapy

Although autologous CAR-T cell therapy for hematopoietic cancer is safe and effective, there are some limitations such as the long period to prepare sufficient amounts of CAR-T cells to be injected. Therefore, allogeneic therapy has been robustly tested and showed promise in some studies. Early trials to see the effectiveness and safety of allogeneic therapy with CAR-T cells has been done with donor-derived CD19-targeted T cells, and showed minimal residual disease-negative remission in patients with relapsed B-ALL after HSCT [39]. However, the use of donor CAR-T cells has the issue of potentially inducing alloreactivity, and from 132 patients with B-cell malignancies, 4% and 3% of the patients developed acute GvHD and chronic GvHD, respectively, [40]. In addition, since autologous CAR-T cell therapy showed significant toxicity issues from clinical trials, toxicity derived from allogeneic CAR-T cells is also predicted to be significant [41]. Nevertheless, allogeneic CAR-T cells therapy has been applied to many patients because of the great advantage of being available off-the-shelf. The source of CAR-T cells can be autologous or allogeneic. Allogeneic source of CAR-T cells is seen in patients that have allogeneic hematopoietic stem cell transplantations and can be either donor-derived or recipient-derived. In this study, recipient-derived CAR-T cell therapy showed an increased complete remission (CR) rate with less severe CRS patients than autologous CAR-T cell therapy. Even though recipient-derived CAR-T cells are not truly allogeneic, this study reveals the potential therapeutic effect of allogeneic CAR-T cells therapy for patients with relapsed/refractory ALL [42]. Meanwhile, post allogeneic HSCT was tried after CAR-T cell therapy for patients with ALL, NHL (B-cell non-Hodgkin lymphoma), and CLL (chronic lymphocytic leukemia) to evaluate posttransplant toxicities. In this study, ALL patients received post allogeneic HSCT and NHL/CLL patients received myeloablative conditioning, but showed higher mortality [43].

## 4. GvHD Associated with Allogeneic CAR-T Cell Therapy

GvHD has always been associated with an issue that needs to be overcome or minimized when allogeneic CAR-T cell therapy was applied. Early studies revealed that donor origin CAR-T therapy for patients with B cell associated disease showed very low levels of GvHD [44,45,46,47,48] even though there were toxicity issues such as hypotension and fever [46]. In addition, a mouse study to investigate the effect of donor-derived CD19 CAR-T cells in allogeneic HSCT showed that allogeneic CAR-T cells exert potent graft-versus-lymphoma activity and decreased GvHD and identified that TNFRSF9 co-stimulated CAR-T cells increased the occurrence of GvHD [49] even though TNFRSF9 co-stimulation has been known to decrease T cell exhaustion by repetitive CAR signaling [50,51]. However, using allogeneic CAR-T cells obtained from either an HLA haploidentical or completely mismatched healthy donor showed grade 3 hematological toxicity with GvHD manifestation [52]. Furthermore, 25% acute or 10% chronic GvHD occurrence were reported in a study with ALL, NHL, and CLL patients [43]. Therefore, allogenic CAR-T cell therapy requires further technological advances to overcome GvHD.

## 5. Engineering Allogeneic CAR-T Cells

Avoiding GvHD is a major hurdle to overcome in allogenic CAR-T cell therapy, especially when the CAR-T cells applied to the patient are generated from HLA mismatched healthy donors.

### 5.1. TR Deletion

The majority of GvHD occurs as a consequence of the diverse repertoire of the allogeneic T cell receptor (TR). To overcome this issue, new engineered CAR-T cells on TR were developed using various techniques. For example, CD19-specific CAR-T cells without endogenous expression of TR was created using designer zinc finger nuclease (ZFN) systems harboring CD19-specific CAR that uses the Sleeping Beauty transposon/transposase system [53]. More efficient CAR-T cells were also generated by disrupting both TR and beta-2 microglobulin that are responsible for GvHD and Host-versus-Graft Effect (HvGE), respectively, as well as by disrupting binding of PDCD1 (PD1) to CD274 (PDL-1), and thereby increased effectiveness of CAR-T cell therapy [54]. Recently, advanced multiplex engineering of T cells using CRISPR/Cas9 without double strand break-induced translocations was developed and showed improved expansion of engineered T cells and reduced safety risks associated with unintended genomic alterations and genotoxicity [55]. Engineering CAR-T cells without rearrangement of endogenous TR locus was accomplished with T cells expressing tumor specific T cell receptor (TR). Mono-specific TR-transgenic cells lacking endogenous rearrangements can be generated from cord blood hematopoietic progenitor cells (HPCs), resulting in a lack of expression of the endogenous TR/CD3 receptor, but showing anti-tumor activities [56,57]. In addition, to generate CAR-T cells targeting a specific antigen, the endogenous TR locus was replaced with a CAR construct using homing endonuclease and an Adeno-associated virus (AAV) donor template that allowed for the expression of CAR under the endogenous TR promoter [58].

Another approach to shut down the signaling pathway triggered from TR was attempted by transducing CD247-binding single-chain variable fragment (scFv) with an ER retention signal and a transmembrane domain. This CD247-specific scFv overexpressed in the ER could capture CD247 and thereby inhibit the interaction of CD247 with TR, resulting in inhibition of TR signal transduction [59]. More advanced allogeneic CAR-T cells were created by adding safety options. TR depleted CAR-T cells generated through the transcription activator-like effector nuclease (TALEN) technique were further transduced with a CD20 mimotope (antigen-mimicking peptide) expressing gene. In this system, overactivated CAR-T cells could be targeted by rituximab that recognizes the CD20 mimotope and blocks activation signal, and thereby the cells are eliminated [60]. This is especially useful in reducing the death rate induced by cytokine releasing syndrome during CAR-T therapy.

### 5.2. Potential Inhibitors to Prevent GvHD

**Figure 2:**
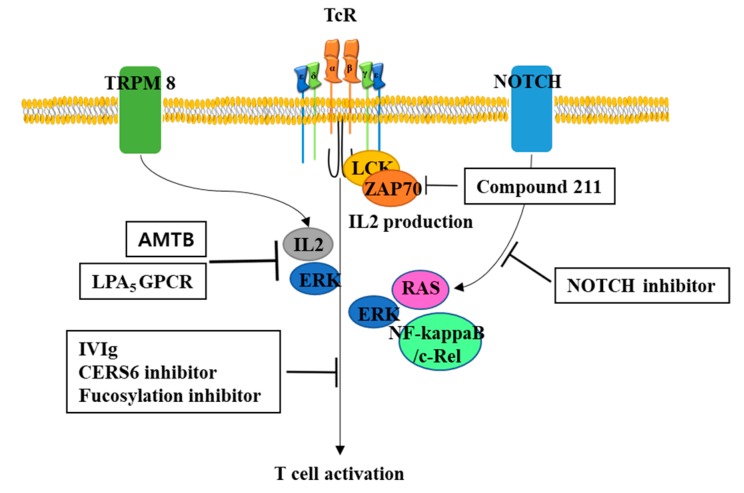
Potential inhibitors for T cell activation. To minimize GvHD, inhibitors for T cell activation may be useful. Intravenous immunoglobulin (IVIg), ceramide synthase 6 (CERS6) inhibitor, fucosylation inhibitor have known to inhibit T cell activation, which may be used to ameliorate GvHD during CAR-T cells therapy. Another potential inhibitor could be N-(3-aminopropyl)-2-[(3-methylphenyl)methoxy]-N-(2-thienylmethyl)-benzamide hydrochloride (AMTB) and Lysophosphatidic acid G protein coupled receptor (LPA_5_ GPCR), which have known to inhibit calcium-mediated T cell activation. Compound 211, allosteric noncompetitive selective inhibitor of CD45 tyrosine phosphatase, could inhibit TR-mediated expression and activation of LCK, ZAP70, ERK, and IL2. Inhibitors for Notch signaling could be used to decrease T cell activation by decreasing activity of RAS (H-, K-, N-), ERK, and NF-kappaB.

Another possible approach to inhibit the allogeneic GvHD effect of CAR-T therapy is to treat with agents inhibiting the TR signaling pathway as summarized in Figure 2. Recently, intravenous immunoglobulin (IVIg) that is generally used for treatment of autoimmune and infectious disorders was identified to inhibit the signaling pathway mediated by TR [61]. Lysophosphatidic acid (LPA_5_) G protein coupled receptor (GPCR) was identified to be expressed in CD8 T cells and to engage the lysophosphatidic acid bioactive serum lipid that is normally increased in chronic inflammatory disorders, thereby suppressing early TR signaling including calcium mobilization and ERK activation [62,63]. These suggests that a captured antibody for LPA in the serum could be used to decrease GvHD mediated by TR. High fucosylation has been known to occur in TR and is known to be related to TR activation [64,65,66]; therefore, co-treatment with fucosylation inhibitor to reduce TR signaling seems promising. Calcium channel inhibitor N-(3-aminopropyl)-2-[(3-methylphenyl)methoxy]-N-(2-thienylmethyl)-benzamide hydrochloride (AMTB) has been identified to inhibit the transient receptor potential melastatin (TRPM) 8 channel and suppress murine T cell activation by inhibiting IL12 expression and activation of the ERK signaling pathway [67].

An allosteric noncompetitive selective inhibitor of CD45 tyrosine phosphatase, Compound 211, also prevents TR-mediated expression and activation of LCK, ZAP70, ERK, and IL2 in cultured primary T cells [68]. Ceramide synthase 6 (CERS6) has been known to be required for optimal T cell activation, proliferation, and cytokine production. In a trial to see the relationship between GvHD development and CERS6 activation, a specific inhibitor for CERS6 was applied to murine and human T cells and showed significantly reduced T cell activation, suggesting that the CERS6 inhibitor may be a promising reagent to control GvHD [69]. Furthermore, small molecule c-Rel inhibitor showed reduced alloactivation of T cells without affecting anti-tumor activity by impairing negative feedback on IL2 production [70]. Notch inhibition has also showed anti-GvHD effects induced in CD4+ or CD8+ T cells and showed decreased RAS/ERK and NF-kappaB activity upon re-stimulation through the TR [71].

Overall, various trials have been conducted to diminish GvHD through genetic engineering, mainly by TR inactivation and specific inhibitors mediated by TR signaling. It is of course important to decrease the allogeneic GvHD effects of CAR-T cells, but before being applied to patients, careful validation is required to determine whether the CAR-T cells can mediate toxicity through genetic engineering or inhibitors.

## 6. Universal CAR-T by Switch Molecules for Allogenic Application

More powerful and universal use of allogeneic CAR-T cells could be achieved by adding switch molecules between CAR-T cells and tumor cells, named universal allogeneic CAR-T cells (UniCAR-T). Specific switch molecules that are responsible for linking CAR construct of CAR-T cells to antibody-recognizing TAAs in tumor cells allow for universal use of CAR-T cells for targeting various tumor cells. Switch molecules could be created by using protein–protein interaction or protein–chemical interaction in which each part should be connected to either the antibody or CAR. Therefore, the UniCAR-T system has the great advantage of controlling CAR-T cell activity by changing the amount of switch molecule–antibody complexes to be injected [72]. Various antibodies targeting TAAs could easily be linked to molecular adaptors, either chemically or genetically. For example, an antibody-based bifunctional switch was developed, structured with a peptide neo-epitope (PNE) connected to the tumor antigen-specific Fab region targeting TAA and it could bind to the anti-PNE scFv that is connected to CAR. This switch model allows for control of activity, tissue-homing, cytokine release, and phenotype of CAR-T cells in a dose dependent manner [73,74]. Another type of switch molecule is the FITC–folate system. Instead of using an antibody-recognizing TAA, folate, which recognizes the folate receptor alpha (FOLR1) receptor that is normally overexpressed on ~40% of human cancer cells such as breast, lung, uterus, and ovarian [75,76,77], was linked to FITC, which could then be recognized by the anti-FITC antibody connected to CAR on the CAR-T cells [78,79,80]. Therefore, tumor cells overexpressing FRα is recognized by folate that is linked to FITC, and then FITC is recognized by the anti-FITC antibody, resulting in CAR-T cell activation. The potential therapeutic effect of this folate/anti-FITC system in childhood solid tumor was evaluated in preclinical trial [81]. Indeed, the binding affinity of the CAR for FITC was measured as Kd = 30 pM (3 × 10^−11^ M) and the affinity of the folate for FRα receptor on MDA-MB-231 cells was determined to be Kd = 1.0 nM [82]. Anti-La 5B9 mAb that recognizes a continuous sequence of 10 amino acids (5B9 tag) of the nuclear protein La/SS-B was used as a switch molecule that was linked to CAR and the 5B9 tag was linked to scFv recognizing TAA [83]. In addition, FITC-HM-3 bifunctional molecule (FHBM) was created as a switch molecule for novel switchable dual-receptor CAR-engineered T cells. Two CARs are created and each CAR has the ability to bind to TAA or FITC of FHBM, respectively. Because each CAR has a CD247 and TNFRSF9 domain as a signaling inducer inside the cell, both CARs should be activated. So, one CAR should bind to TAA and another CAR should bind to FITC of FHBM for CAR-T cell activation [84]. This dual activation system with a switch molecule could efficiently control CAR-T cells’ activity.

Another switch molecule is a leucine zipper domain, which is an endogenous nuclear molecule interaction domain widely involved in protein–protein interaction. The SUPRA (split, universal, and programmable) CAR system utilizes a leucine zipper domain as a switch molecule [85]. Leucine zipper dimerization system is a common structure that has a leucine amino acid at every 7th position and forms an alpha-helix structure, allowing for strong interaction between proteins harboring leucine zipper domains. The interaction strength and direction (heterodimerization or homodimerization) of a leucine zipper domain determines the types of amino acids near the leucine positions, thus allowing for easy designing to control the interaction specificity between leucine zipper domains [86,87,88]. The binding affinity of leucine zipper could theoretically be up to Kd = 10^−15^ M [89,90], but so far has been reported to be Kd = < 10^−8^ M by genetic engineering [91].

**Figure 3:**
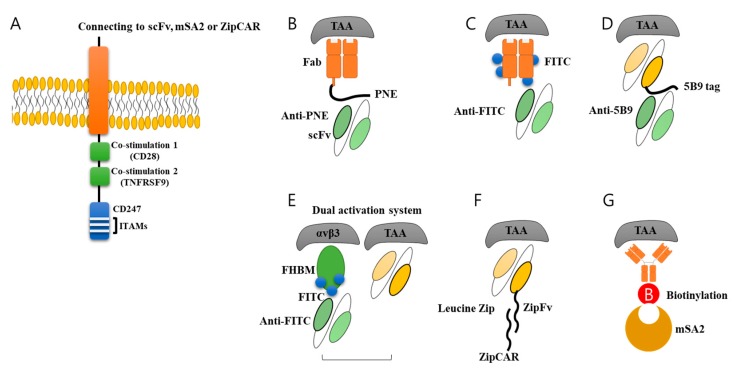
Various switch molecules. For universal use of CAR-T cells, various switch molecules were developed. (**A**) General CAR structure is composed of signaling domains inside cell, transmembrane domain, and scFv outside cells. The scFv part could be modified further by adding switch molecules. (**B**) Peptide neo-epitope (PNE), (**C**) fluorescein (FITC), (**D**) 10 amino acids (5B9 tag), (**E**) FITC-HM-3 bifunctional molecule (FHBM) and scFv, and (**F**) Leucine ZipFv linked to antibody, and (**G**) Streptavidin 2 (mSA2) biotin-binding domain was developed to be used as switch molecule. These molecules could be recognized by antibody or ZipCAR connected to CAR, resulting in induction and activation of signaling pathway in CAR-T cells. This system has great advantage of controlling CAR-T cell activity by changing the amount of switch molecules linked to antibody.

Monomeric streptavidin 2 (mSA2) biotin-binding domain was also introduced as a switch molecule. The biotinylated antibody that recognizes TAA could be captured by a CAR-T cell with an mSA2 domain linked to the CAR structure [92]. Switch molecules described in this text are summarized in Figure 3.

During development, the switch molecules should be chosen such that they do not induce additional immunogenic responses such as GvHD and their specificity should ensure minimal reactivity.

## 7. Safety System for CAR-T Cells

Although the effectiveness of CAR-T therapy has been improved, there are still side-effects such as neurotoxicity and CRS that can still be observed when applied to patients. Here, we summarized what new techniques have been introduced to minimize toxicity and eventually increase efficiency of CAR-T therapy. One of the safety switches is using a kinase inhibitor that blocks the signaling pathway mediated by TR. Dasatinib, which is a well-known inhibitor for lymphocyte-specific protein tyrosine kinase (LCK), could block phosphorylation of CD247 mediated by LCK, thereby ablating the signaling pathway mediated by CARs containing either CD28 plus CD247 or TNFRSF9 plus the CD247 activation domain [93]. Another method to safely activate CAR-T cells is to use a suicide-gene-inducible system. Overactivated CAR-T cells could be eliminated by rimiducid-inducible MyD88 and CD40 activation. In this system, injection of rimiducid induced caspase-9-dependent CAR-T cell apoptosis, enhancing cell proliferation, cytokine secretion, and anti-tumor efficacy [94]. Another suicide gene is Herpes Simplex Virus thymidine kinase (HSV-TK), which is inserted into the CD44 isoform variant 6 (CD44v6) CAR plasmid and conditionally co-expressed when needed [95]. Rituximab has been used to treat many cancers, such as non-Hodgkin’s lymphoma, rheumatoid arthritis, and chronic lymphocytic leukemia, by targeting CD20 on the B-cells of the related disease [96,97,98,99,100]. By creating CAR-T cells overexpressing CD20, rituximab treatment could be used as a safety reagent when CAR-T cells are overactivated. This is a very rapid control method to reduce cytokine release, and can even result in CAR-T cell death [101,102]. A truncated version of the epidermal growth factor receptor (EGFRt) was also expressed as a safety suicide gene in CD19 CAR-T cells and could be targeted by cetuximab. Cetuximab treatment showed efficient elimination of CD19 CAR-T cells both early and later after adoptive transfer in mice [103]. Various safety systems to regulate CAR-T cell activity are still being developed, but it is important to predict the potential side-effects and should be tested before being fully applied to patients.

## 8. Conclusions

Known as a living drug, CAR-T cell therapy, used to treat patients with B-ALL without improvement with other known therapies such as HSCT and chemotherapies, has been performed intensively over the recent decade and has showed promising results. Yet YESCARTA and KYMRIAH were the only FDA approved CAR-T cells used to treat adult patients as a last resort after all other treatments. There are still many obstacles to overcome so as to shorten the re-injection time for patients when using autologous CAR-T cell therapy. For those patients that do not have easily available autologous T cells and for low-cost universal CAR-T cells, the development of allogenic universal CAR-T therapy is in high demand. In the CAR-T therapeutic protocol, it is essential to minimize GvHD by adding safety system. Many technical advances such as gene editing have been made to solve these problems. In addition, recent studies have focused on the use of CAR-T cells in solid tumors, demonstrating an expanded use of CAR-T cell therapy and opening the possibility for its use in other diseases such as metabolic, heart, and rare diseases in the near future.
